# NMR Metabolomics for Stem Cell type discrimination

**DOI:** 10.1038/s41598-017-16043-8

**Published:** 2017-11-17

**Authors:** Franca Castiglione, Monica Ferro, Evangelos Mavroudakis, Rosalia Pellitteri, Patrizia Bossolasco, Damiano Zaccheo, Massimo Morbidelli, Vincenzo Silani, Andrea Mele, Davide Moscatelli, Lidia Cova

**Affiliations:** 1 Department of Chemistry Materials and Chemical Engineering “G. Natta”, Politecnico di Milano, Milan, Italy; 2 Institute of Neurological Sciences, CNR, Section of Catania, Catania, Italy; 3 Department of Neurology and Lab. Neuroscience, IRCCS Istituto Auxologico Italiano, Milan, Italy; 4 Department of Experimental Medicine, section of Human Anatomy, University of Genoa, Genoa, Italy; 5 Institute for Chemical and Bioengineering, ETH Zurich, Zurich, Switzerland; 6 Department Pathophysiology and Transplantation - “Dino Ferrari” Center, Università degli Studi di Milano, Milan, Italy; 7 ICRM Istituto di Chimica del Riconoscimento Molecolare, CNR, Milan, Italy; 8 Present Address: Environmental Research Laboratory, National Center for Scientific Research “Demokritos”, Agia Paraskevi Attikis, 15310 Greece

## Abstract

Cell metabolism is a key determinant factor for the pluripotency and fate commitment of Stem Cells (SCs) during development, ageing, pathological onset and progression. We derived and cultured selected subpopulations of rodent fetal, postnatal, adult Neural SCs (NSCs) and postnatal glial progenitors, Olfactory Ensheathing Cells (OECs), respectively from the subventricular zone (SVZ) and the olfactory bulb (OB). Cell lysates were analyzed by proton Nuclear Magnetic Resonance (^1^H-NMR) spectroscopy leading to metabolites identification and quantitation. Subsequent multivariate analysis of NMR data by Principal Component Analysis (PCA), and Partial Least Square Discriminant Analysis (PLS-DA) allowed data reduction and cluster analysis. This strategy ensures the definition of specific features in the metabolic content of phenotypically similar SCs sharing a common developmental origin. The metabolic fingerprints for selective metabolites or for the whole spectra demonstrated enhanced peculiarities among cell types. The key result of our work is a neat divergence between OECs and the remaining NSC cells. We also show that statistically significant differences for selective metabolites characterizes NSCs of different ages. Finally, the retrived metabolome in cell cultures correlates to the physiological SC features, thus allowing an integrated bioengineering approach for biologic fingerprints able to dissect the (neural) SC molecular specificities.

## Introduction

Stem cells (SCs) are undifferentiated cells in organisms, which could indefinitely proliferate or generate differentiated cell types able to perform specific physiological functions. Their organized proliferation and cell fate determination allow the formation of both tissues and organs during embryonic development, whereas their permanence in restricted niches consents tissue repair and maintenance in adulthood^1^. Therefore, SCs are currently used in several biomedical applications, especially to replace or repair pathological tissues in regenerative medicine, as well demonstrated for amyotrophic lateral sclerosis^2^. Stem cell cultures appear as a heterogeneous mixture of functional subpopulations, characterized by distinct self-renewal and differentiation biases, able to generate a progressively restricted repertoire of cell types (progenitor cells)^3^. Heterogeneity is also high in other SC types, such as pluripotent SCs, Embryonic Stem Cells (ESCs) and induced pluripotent stem (iPS) cells, so their application to cell therapy requires accurate selection of pure SCs populations^3^. To fulfill safety criteria for regenerative medicine^4^, the absence of residual undifferentiated SCs and preparations of pure standardized SCs should be guaranteed, as requested by the International Society for Stem Cell Research (ISSCR)^5,6^.

In this general context, the determination of (univocal) biomarkers able to define SC/cell line identities and functions along passages plays an emerging key role for validating scientific studies^7^, as well as clinical trials or cell therapy products^1^.

Recently, a pivotal role of metabolic pathways has been demonstrated in dictating SC fate since each cell metabolomic profile appears directly influenced by its proliferative or differentiative state^8^, as well as by the surrounding environment. Therefore, Nuclear Magnetic Resonance (NMR)-based metabolomic analysis^9,10^ is constantly gaining importance in the study of SC biology and for the discovery of biomarkers *in vitro* or directly in the Central Nervous System (CNS)^11–15^. Interestingly, alterations of metabolic demands has been also linked to ageing, (neuro)degenerative diseases^16^ and cancer^17^ with potential clinical application to improve diagnostic accuracy, define prognosis as well as predict and monitor treatment efficacy^18,19^. Robustness and reliability of quantitative analysis by proton NMR (^1^H-NMR) in metabolomics is supported by several reports wherein SC normal/tumor subtypes or their conditioned media may be efficiently distinguished by specific NMR patterns, as well as metabolites^12,20,21^. In this general scenario it should be pointed out that the possible use of standard NMR spectrometers and a sample preparation without any special requirement (e.g. the use of ^13^C-hyperpolarized initiators) is considered a plus for metabolomic profiling^22,23^.

Actively proliferating Neural Stem Cells (NSCs) were here compared to a selected glial subpopulation, the progenitor Olfactory Ensheathing Cells (OECs), to test the reliability of ^1^H-NMR technique in providing effective biomarkers for cell discrimination. NSCs are undifferentiated multipotent SCs located in the SubVentricular Zone (SVZ) of adult mammalians and able to generate differentiated both neuronal and glial progeny through intermediate progenitors^24^. Multipotent NSCs are present all along brain development: in embryonic brains they are mainly involved in tissue formation and neuron production, whereas later (P1) they participate to the functional development and remodeling of brain’s connections. Finally, in adult life NSCs remain quiescent only in specific brain areas and can be re-activated “on demand” to generate new neurons functionally integrated into the adult mammalian brain^24^. Therefore, NSCs shift their main commitment *in vivo* from creation of a brain neural network (E12) and its refinement (P1) to physiological (single) cell substitution in damaged or aged brain tissue. NSCs can be isolated from embryonic/postnatal or adult brains and maintained *in vitro*, but they are undistinguishable by cellular phenotype, differentiative potential as well as antigenic markers^25,26^.

More committed glial-like OECs share with NSCs the embryonic SVZ origin, but mainly support healthy neuronal homeostasis in the olfactory system. Physiologically, OECs are specific glial progenitor cells enveloping the olfactory nerve which derive from the migratory stream connecting the SVZ to the olfactory bulb (OB) (see Supplementary Fig. SI1). They are able to remyelinate demyelinated axons as well as to support regrowth of transected axons after transplantation into the adult CNS^27^. Although NSCs and OECs are functionally different, they both express nestin and share a common embryonic derivation (see Supplementary Fig. SI1). We derived neuronal progenitor cultures from mice brains of: i) fetal (E12), ii) postnatal (P1) and iii) adult (AD) NSCs from the SVZ and iv) postnatal OECs from the OB of P1 mice (see Supplementary Fig. SI1).

In the present work the identification of specific metabolic markers in cultured brain SC types and the comparative application of NMR analysis coupled to multivariate analysis for a rapid and easy assessment of stem cell peculiar metabolome are reported.

The definition of biological hallmarks/biomarkers and the characterization of phenotypically similar SC types by NMR analysis are thus the final goals of the present study.

The metabolic profile of the different SC types was acquired and analyzed via ^1^H NMR spectroscopy through the following steps: i) identification and quantitation of the SC metabolites obtained from the cell cultures by ^1^H-NMR spectroscopy, ii) discovery of selective and statistically significant metabolic markers characterizing SCs, with focus on the specific profile of OECs in respect to NSCs, and iii) the clustering and differentiation of the SC types on the basis of their metabolic profile via multivariate analyses^28,29^– Principal Component Analysis (PCA) and Partial Least Square Discriminant Analysis (PLS-DA) – of the entire ^1^H-NMR spectra.

## Results

### Identification of metabolites via ^1^H NMR Spectroscopy

Analysis of ^1^H NMR metabolite was performed on the four different, but related, SC types (see Supplementary Fig. SI1) on several independent replicates derived from different primary cultures grown in the specific media for several passages, as detailed in experimental section.

**Figure 1:**
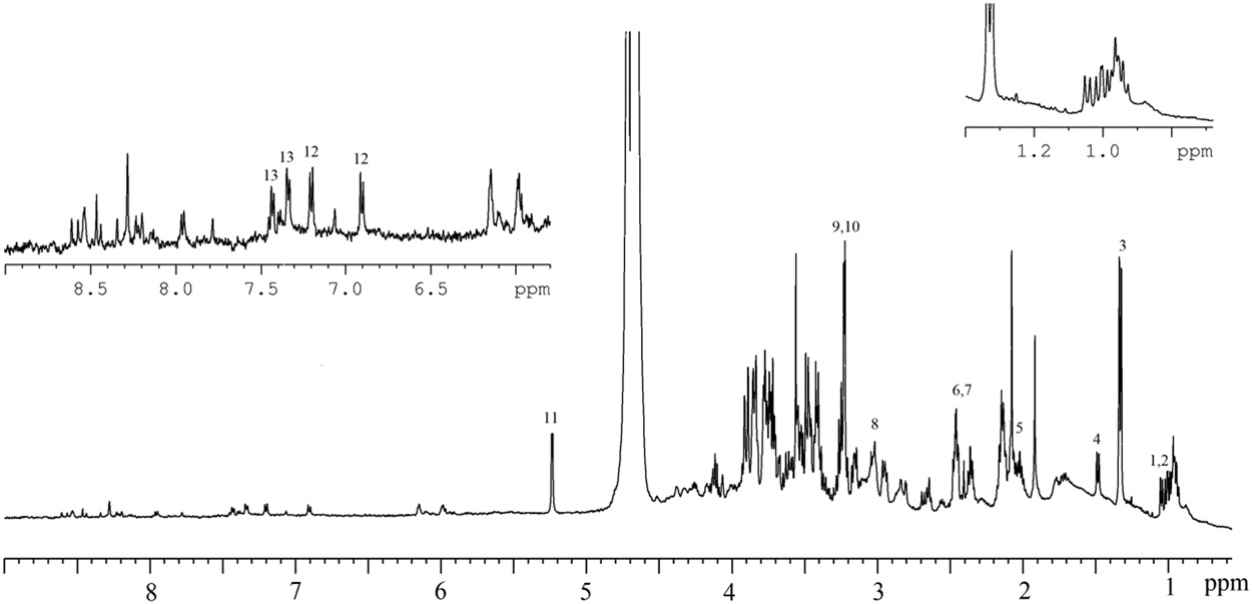
Representative ^1^H NMR spectrum of AD NSC lysate. Peak assignment of specific metabolites is indicated as: (1) Leucine (Leu), (2) Isoleucine (Ile), (3) Lactate (Lac), (4) Alanine (Ala), (5) Acetate (Ace), (6) Glutamate (Glu), (7) Succinate (Suc), (8) Creatine, (9) Taurine (Tau), (10) Choline (Cho), (11) Glucose (Glc), (12) Tyrosine (Tyr), (13) Phenylalanine (Phe).

**Figure 2:**
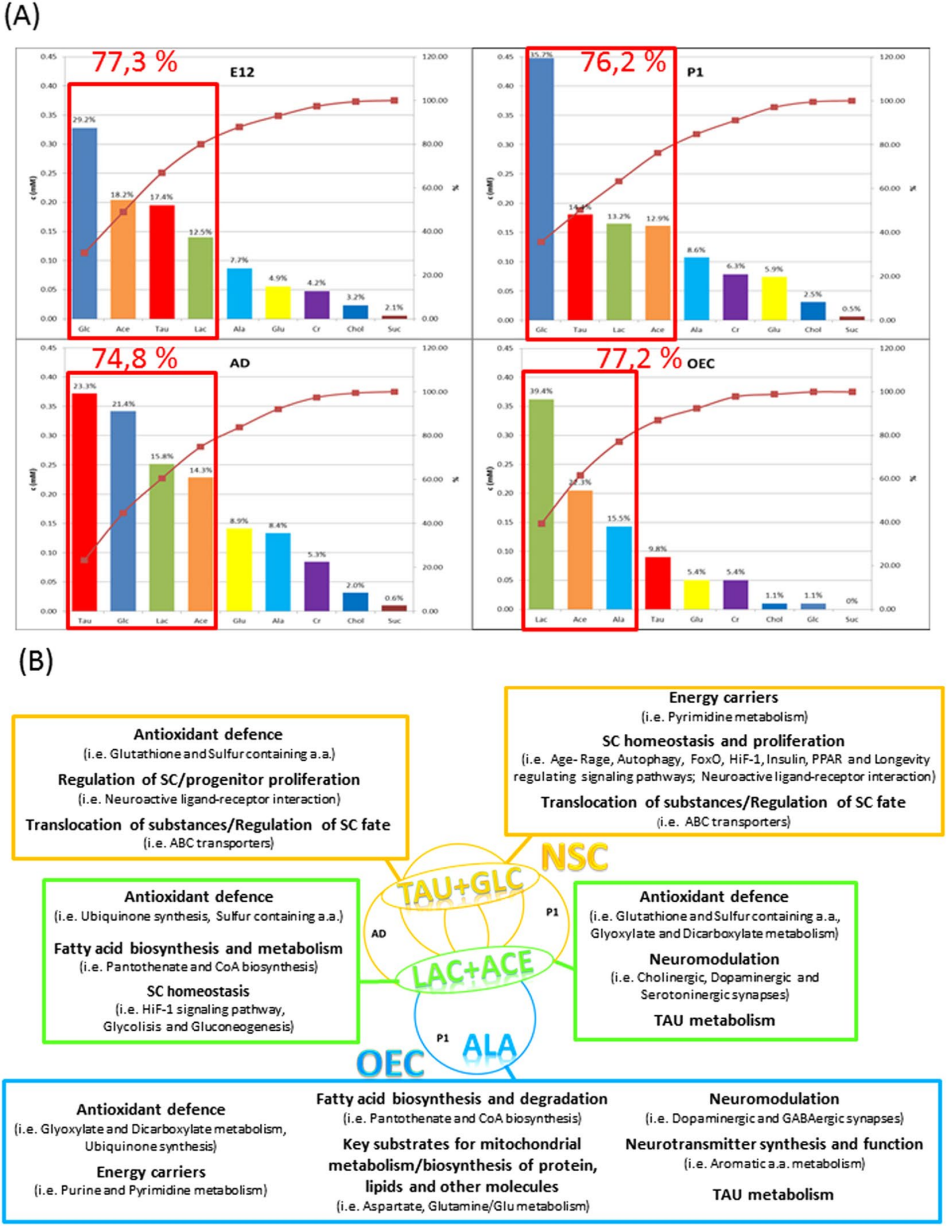
More representative metabolites for each cell type and the related biological pathways. (**A**) Pareto analysis of main retrieved metabolites’ concentration for each cell type. The histograms report the bars in decreasing order for each cell type. The curve on the plot accounts for the cumulative percentage. The corresponding values are reported on the secondary y-axis. (**B**) The analysis of more nine representative metabolites in each cell type reveals that both common (Lac, Ace) and differential metabolites (Tau and Glc for NSCs; Ala for OECs) comprise for more than 74% of total metabolome. A search in the Kegg pathway database reveals main biological topics involving the different metabolites: all NSCs showed higher Tau and Glc levels whereas larger Lac amounts characterized OECs. More details on all the involved biological pathways analyzed are reported in Supplementary Fig. S5. Abbreviations: ABC transporters = ATP-binding cassette transporters; Aromatic a.a. = aromatic amino acids (Tyrosine, Triptophan, Phenylalanine); PPAR = Peroxisome proliferator-activated receptors; HIF-1 = Hypoxia-inducible factor 1; FoxO = Forkhead box O transcription factors; SC = Stem Cells; Sulfur containing a.a. = Sulfur containing amino acids.

The metabolites were extracted for each replicate, as described, afterwards the corresponding ^1^H-NMR spectrum was acquired and analyzed. An example of a full ^1^H NMR spectrum for the metabolic profile of the SCs along with the assignment of the main identified metabolites is shown in Fig. 1. The overlay of the ^1^H NMR spectra of all the SCs examined is reported in Supplementary Fig. SI2. It is worth mentioning the fact that the specific OEC metabolic fingerprint via NMR spectroscopy on cultured cells is presented here for the first time. The ^1^H NMR profiles of all the SCs examined shared the same set of metabolites, although with different peak intensities. Detailed spectral assignment were supported by the analysis of the two-dimensional ^1^H Total Correlation Spectroscopy (TOCSY) NMR experiments (Supplementary Fig. SI3). The SC metabolome includes different biochemical categories, as shown in Figs 1 and 2: non-aromatic amino acids, such as Alanine (Ala), Leucine (Leu), Isoleucine (Ile) and Glutamate (Glu, essential for biosynthesis and anaplerosis), di-carboxylic acids, such as Succinate (Suc, which plays a crucial role in adenosine triphosphate (ATP) generation in mitochondria) and energy metabolism components, mainly Glucose (Glc). Only the anomeric signal of α-D-Glucopyranose (here indicated as Glc) was observed at 5.24 ppm, whereas the corresponding signal for the β-anomer was hidden by the water peak. Additionally, the presence of the metabolites characteristic of healthy brain (such as total Creatine (Cr) and Choline-containing compounds (Cho), both related to enhanced cell membrane turnover in rapidly dividing cells) in conjunction with Lactate (Lac) from glycolysis were also observed. Additional categories were retrieved, including antioxidants, such as Taurine (Tau), and other metabolites related to energy homeostasis, such as Acetate (Ace) as well as Alanine (Ala)^1,14,30,31^. Finally, the part of the ^1^H NMR spectrum at high ppm values (low field) showed small but significant peaks consistent with aromatic protons. Some signals could be unambiguously assigned to aromatic amino acids, such as Tyrosine (Tyr) and Phenylalanine (Phe) (see Fig. 1). Such peaks were detected, with variable intensity, in the majority of SC samples. The other aromatic peaks visible in Fig. 1 could not be assigned and will not be further discussed.

### NMR profiling of each SC type

Quantitative evaluation of metabolites concentrations was performed by integration and subsequent normalization of specific peaks (see Fig. 1). The histogram of the main metabolite concentrations is shown in Supplementary Fig. SI4 and the corresponding Pareto analysis for each cell type is presented in Fig. 2A. In Fig. 2B more representative measured nine metabolites (around 75% of the analysed metabolites) are depicted with indication of the correlated main metabolic pathways. Some metabolites were specifically abundant in all NSCs (Tau and Glc) or only in OECs (Ala) whereas Lac and Ace were considerably present in all cell types. The complete list of all metabolic Kegg pathways related to the metabolites is reported in Supplementary Fig. SI5.

The goal of a quantitative, significant test based on the metabolic profile was achieved by comparing the single metabolite variation among cell types. The difference in the metabolite concentration was tested by one-way ANOVA test in search of selective markers for the discrimination of cell types.

**Figure 3:**
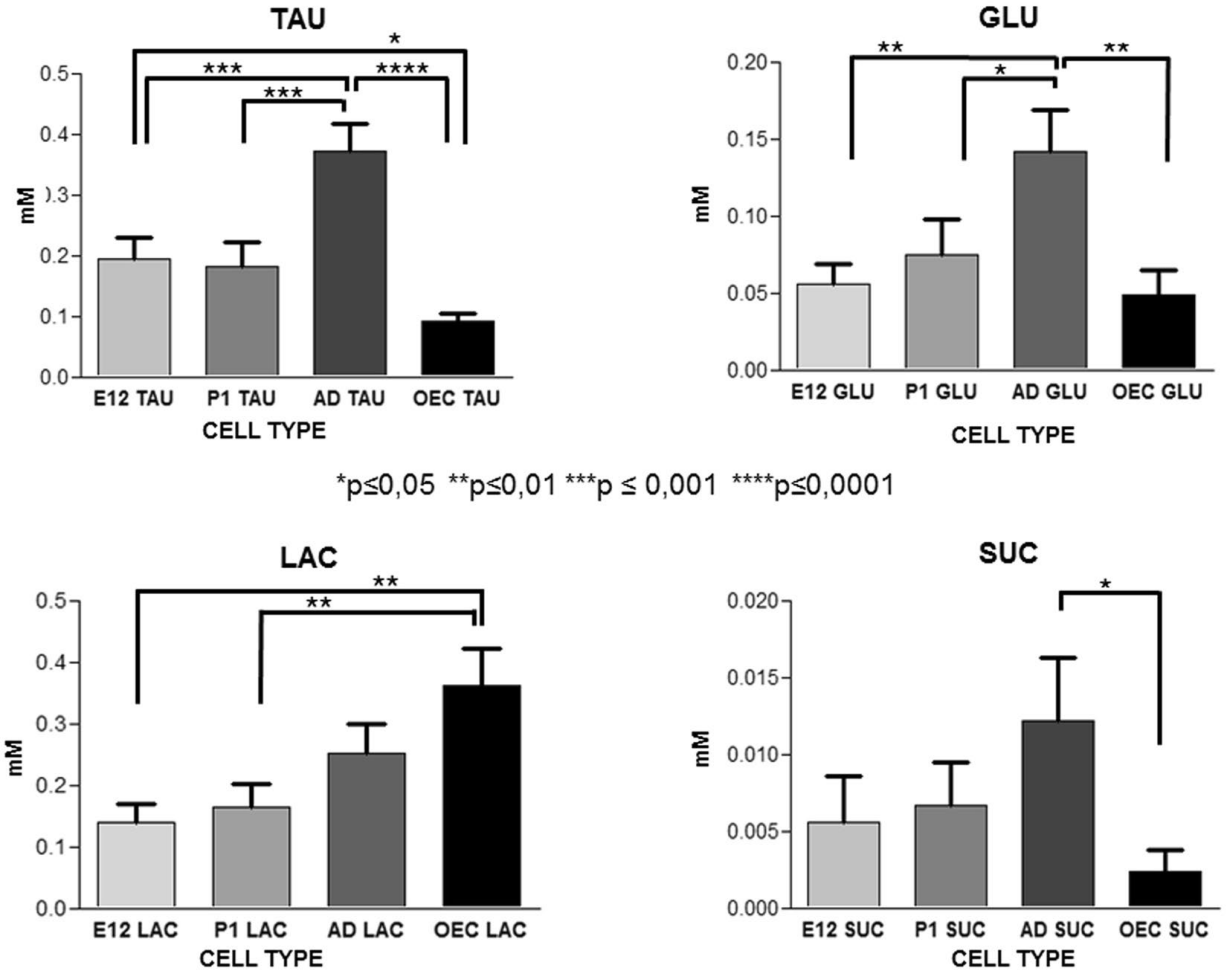
Comparison among metabolite concentrations. Each set of graphic reports on the different level of the titled metabolite in the four cell subtypes tested by one-way ANOVA statistical analysis. The connectors on top of the bars indicate the statistically significant differences and are reported only in the cases of significant difference. Statistical significance is indicated in each graph as ***ppp ≤ 0.001, **pp ≤ 0.01 and *p ≤ 0.05 versus the corresponding sample(s). Error bars are referred to the standard error.

The statistical analysis of the NMR data, shown in Fig. 3, identified Tau, Glu, Lac, and Suc as the set of biological markers for a differentiative analysis of the four SCs. Indeed, these metabolites significantly varies among cells, thus suggesting that the cell metabolome reflects some peculiarities of cell subtypes, irrispectively of a phenotypic and antigenic homogeny. We retrived that significant higher Tau and Glu content characterizes AD in comparison to all other SC subtypes, whereas no significative differences in metabolite contents were always observed when E12 and P1 were compared. Moreover, Lac content significantly varied between E12 or P1 and OECs, whereas intracellular Suc amount efficiently discriminated between AD and OECs wherein the statistically lowest levels were retrieved (Fig. 3). Interestingly, the metabolic contents for these selected markers efficently differentiated OECs from all NSCs and AD from the younger counterparts among NSCs.

The NMR data also pointed out that quantification of the remaining metabolites (such as Cho, Ala, Ace, Cr, and Glc (characterized by a large variability)) did not show statistically significant differences within the examined set of SCs (see Supplementary Fig. SI6). Therefore, we consider these data qualitative and they will not be discussed in the next section. Finally, the Glc content and consumption in the different cell types is reported in Supplementary Fig. SI7 and discussed in Supplementary Information SI section.

### Spatio-temporal cell identification by multivariate NMR analysis

A different and complementary approach to the analysis of NMR data is based on multivariate analyses of the NMR spectra: PCA and PLS-DA. PCA is an unsupervised method able to uncover meaningful patterns in the data set. It provides a representation of the data in a lower-dimensional space, whose coordinates are the principal components (PC).

**Figure 4:**
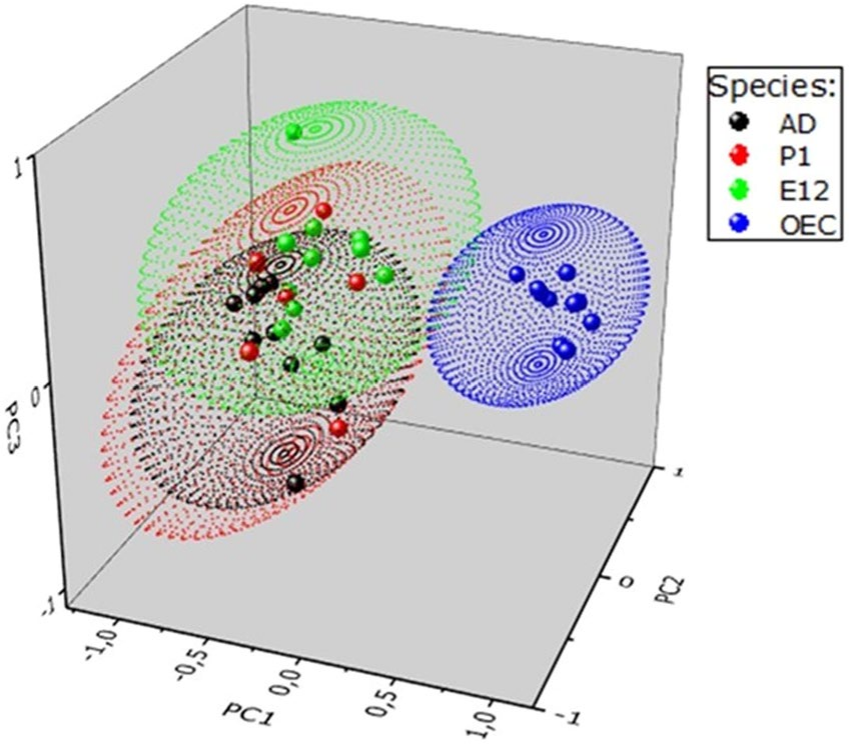
3D Score plot of PLS-DA analysis. The 3D score plot enlightened the similarities and differences among the metabolic fingerprints of each SC type – PLS-DA score plot of PC1 *vs* PC2 *vs* PC3 for: E12, P1, AD, and OEC SCs. The data points correspond to the examined samples. The proximity of two points reflects the metabolic similarity among the corresponding samples. The grouping of each cell type is also outlined by the colour code of the points confined in the ellipsoids. Clustering and separation of the points related to OEC is clearly indicated by the blue ellipsoid. For the presented analysis, the entire ^1^H NMR spectrum was divided in 0.04 ppm regions (bins) for each sample.

PLS-DA is a supervised method introducing a bias regarding the total number of cell types (four in this work) so the PLS-DA model provides better cluster separation. As PCA separation is achieved only when the intra-group variability is remarkably less than the inter-group variability, it provides an unbiased biological indication. Therefore, we used PCA analysis in order to obtain initial, unbiased information on the relationships among the datasets of the SC groups. The results are graphically shown in Supplementary Fig. SI8 in a 3D score plot and, for a better visualization, a two dimensional projection in the PC1-PC2 plane is also reported in Supplementary Fig. SI9A. Both score plots indicate a clear cluster separation between the OECs *vs* all the other SC types. Furthermore, to better sharpen the separation between groups, we carried out the PLS-DA analysis, which is presented in Fig. 4 in the form of the 3D score plot. The scores of cell samples are here mapped in the space by the first three components PC1, PC2 and PC3, which account for 46.3% of the overall variance. The ellipsoids in Fig. 4 show the 95% confidence level and provide a guide for visual observation of cluster separation due to the different metabolic content inside cell subtypes. Similarly to the PCA analysis above, the PC1-PC2 projection of PLS-DA analysis is reported (in Supplementary Fig. SI9B) to provide an alternative view of the cluster separation between OECs and the other SCs. PCA and PLS-DA carried out on the spectral data belonging to the NSC subset only, i.e. by excluding OEC’s datasets, did not allow any cluster separation (see Supplementary Fig. SI10), thus confirming that the results of Fig. 4 and Supplementary Fig. SI8 are driven by the different metabolic profiles of OECs in comparison to the NSC cell types.

Our decision to reduce the number of animals used for our experiments (following the ethical concerns explained at: http://3rs.ccac.ca/en/about/three-rs.html) severely hampered the use of a training set, leading us to the choice of internal cross-validation methods^32^. Therefore, the analysis was cross-validated by the leave-one-out validation protocol. The obtained predictive ability Q^2^ and model fitness R^2^ were 0.44 and 0.66, respectively, thus showing a first evidence of non-overfitting of data. As suggested^33^, this conclusion needs further confirmation by the permutation test. The results in our case for a permutation number = 100 are reported in the Supplementary Fig. SI11 (A), along with the corresponding *p*-value. For clarity, the same test in the case of PLS-DA on NSC subset only is also reported and displayed in Fig. S11(B).

**Figure 5:**
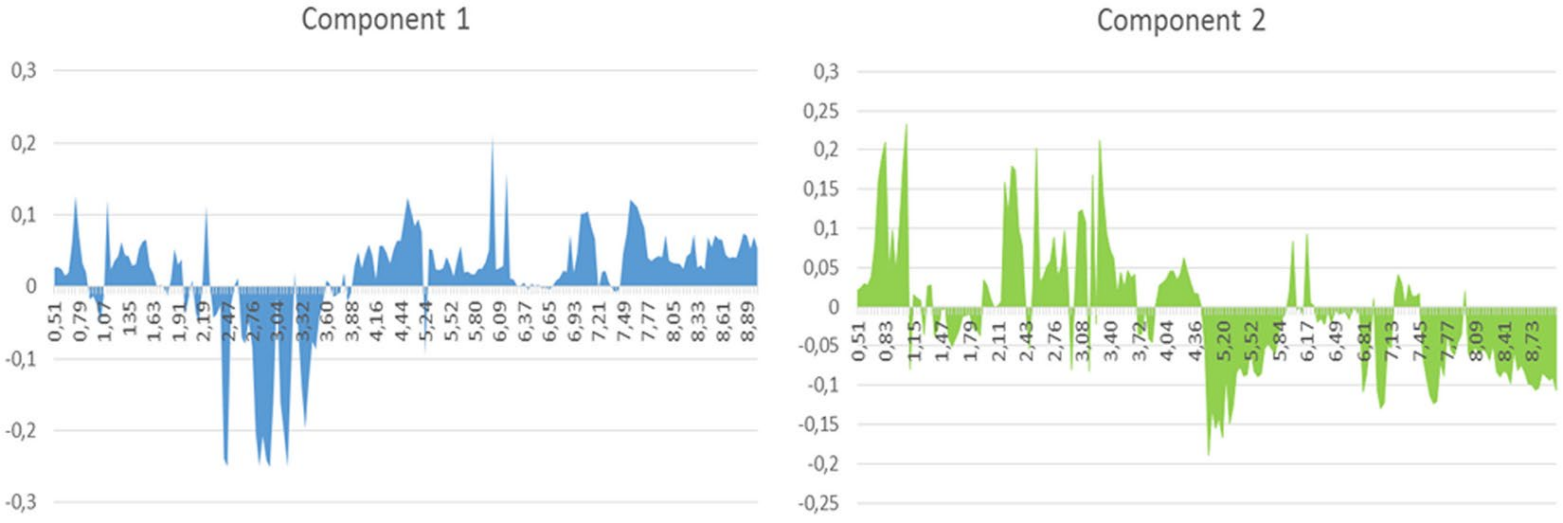
PLS-DA loadings plot of the first two principal component PC1 and PC2 in the one-dimensional representation. The x axis shows the spectral binning with the associated chemical shift values. The y axis values correspond to the relative weight (loading) of the bins.

**Figure 6:**
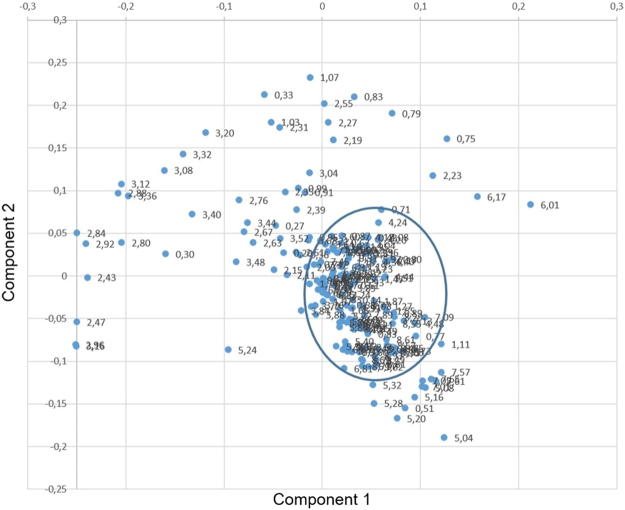
2D loading plot of PLS-DA analysis: PC1 *vs* PC2. In this representation, each point corresponds to a bin of the NMR spectrum in a given chemical shift interval. The ellipse is a guide for eyes to separate clustered and scattered points. The points scattered outside the ellipse are associated to those parts (bins) of the NMR spectrum mostly responsible for the discrimination of SC types observed in the score plot and previously discussed (see Fig. 3 and text). The numbers aside the points correspond to the chemical shift of the bin.

The one dimensional analysis of the loadings for PC1 and PC2 is shown in Fig. 5. The plots illustrate the major contribution to PC1 and PC2 (loadings, y-axes) as a function of the NMR chemical shift (x-axes), thus allowing to identify the NMR signals carrying the major contribution to the spectral differentiation. The results of Fig. 5 indicate that the major contributions to the discrimination of OEC and NSC (AD, P1 and E12) are located in four different regions: from 0.75 to 1.15 ppm, from 2.19 to 3.52 ppm, from 5.04 to 5.36 ppm and from 6.65 to 8.78 ppm. In these spectral regions fall the majority of the signals assigned to Glu, Suc and Tau, which we have highlighted as biological markers on the basis of the statistical analysis reported in the previous section. This plot also point out that even other amino acids, such as Leu and Ile (which have signals that fall respectively at 0.96 and 0.94 ppm), Glc (its H1 signal falls at 5,22 ppm) and some aromatic amino acids (such as Phe) contribute to the separation of clusters.The same data sets can be processed in the form of a two-dimensional PLS-DA loading plot, shown in Fig. 6, where each point represents an individual bin of the experimental spectrum (the “spectrum binning” operation and the meaning of “bin” is detailed in Experimental section at the PCA paragraph). The points of the 2D loading plot of Fig. 6 can be divided into two sets: those inside the crowded region located in the interval 0–0.1 of both PC1 and PC2 dimensions (and highlighted for clarity with an ellipse) and those well scattered outside the crowded region in the PC1-PC2 plane. These latter points are mostly responsible for the cluster separation observed in the score plot of Fig. 4.

The numerical values reported in Fig. 6 are the chemical shift of the corresponding bin. Thus, the loading plots, either in the one- or two dimensional mode (Figs 5 and 6, respectively), can be used in to work out which regions of the NMR spectrum cause the main differences visible in the score plot.

The analysis of the 2D loading plot added information on the bins affording significant contribution to the discrimination among clusters observed in the score plot: it confirms the regions highlighted by the 1D loadings plots showed in Fig. 5.

## Discussion

Our data demonstrate that the metabolic phenotyping of related SCs with similar intracellular content can be achieved by the straightforward application of routine NMR techniques and appropriate statistical analysis. As a matter of fact, selected stem/progenitor populations derived from CNS and maintained in culture mainly share the same metabolites, although in different proportions possibly reflecting preferential biological pathways related to developmental age, stem features and/or biological properties. Therefore, only particular approaches, such as multivariate analysis of NMR data (PCA and PLS-DA), can differentiate these cell types capturing their metabolic peculiarities. Similarly to others (i.e. Pincus & Theriot^34^) we found that the multivariate analysis of the obtained data may contribute to a quantitative description of biologically significant differences in complex systems^35^, such as SCs.

The best distinction was achieved between NSCs and OECs, due to their qualitative and quantitative metabolic differences, both for single metabolite concentrations and elaboration of the complete NMR spectra. Moreover, targeted metabolomics of four metabolites (Tau, Lac, Glu, Suc) clearly identifies some statistical differences between P1 OECs *vs* E12/P1/AD NSCs. Concentrations of Tau and Glu also significantly diverged between AD NSCs *vs* P1 OECs as well as between AD *vs* E12 and P1 NSCs, thus suggesting that some peculiar metabolic features characterize AD NSCs. However, no identified metabolic biomarker allowed a clear distinction between E12 and P1 NSCs. Concordantly, embryonic and postnatal NSCs in the SVZ mainly derive from the same slowly dividing population of neural precursor cells in the embryonic ganglionic eminence^36^, whereas AD NSCs are specifically determined in early embryonic development^37^.

Differently from published literature, our NMR analysis was conducted on selected/pure brain cell subpopulations maintained in cultures: NSCs isolated at different ages and cultured as neurospheres (non-adherent, spherical cultures of clonally derived precursors)^24^; and OECs, a distinct glial cell population with specific biological and molecular properties^38^. Therefore, the NMR profiles we present here are deeply different from the published NMR data on (single) neurons/astrocytic populations^39–42^ as well as from data on glioblastoma stem-like cells/human OB cells^43,44^.

Proteomic analysis on cultured NSCs derived at different ages revealed that the main five canonical pathways highly altered by ageing are related to glycolysis, fatty acid metabolism, propanoate metabolism, protein ubiquitination pathway, and valine, leucine, and isoleucine degradation^25^. Concordantly, around 75% of the nine measured metabolites in our cell populations are related to these biological networks.

The main representative metabolite for both E12 and P1 NSCs is Glc which is related to glycolysis and is essential for modulation of (cell) development, proliferation and differentiation^45^. Concordantly, the Glc content was also consistent in AD NSCs and almost negligible in OECs whose glial fate has been already established.

Additionally, substantial intracellular Tau amounts specifically characterized NSCs, irrespectively of developmental age, but not OECs. The higher levels of Tau retrieved in all NSCs may be related to the key role of this metabolite in antioxidant response, regulation of SC fate/calcium exchange well as neurotransmission/neuromodulation along neural development and neuralization jointed to a neuroprotective and regulative action on apoptotic response^45,46^. The pivotal protective role of Tau in brain physiology is also demonstrated by its altered function in several neurodegenerative diseases wherein contributes to neuronal dysfunction and death^47^.

Essential for the physiological function of NSCs are Tau import^48^ and the ATP-binding cassette (ABC) transporters pathway which is physiologically involved in the membrane trafficking of several substrates (such as ions, sugars, lipids, proteins and drugs^49^) as well as in the regulation of SC biology^50^. Adult NSCs are quiescent cells able to react and proliferate in response to the environmental clues deriving from the surrounding complex network of neurons, microglia, astrocytes and blood vessels^24,45^. Interestingly, Tau levels were comparable between E12 and P1 NSCs, sharing a common embryonic origin^37,48^, whereas the highest content in AD NSCs may be related to a tighter activation/regulation of ABC transporters able to contrast ageing dysfunctions as well as neurodegeneration^51^.

Physiological brain function depends on a constant neuron-glia crosstalk wherein energy reserves and metabolites are actively stored and exchanged between neurons and astrocytes characterized by different, but complementary metabolic specialization^52,53^. In particular, recent literature has focused on the key role of astrocytes both in brain energy metabolism as well as in regulating synaptic neurotransmission^52–54^. Astrocytes are also capable to connect neurons to the surroundings and influence energy supplementation from circulation (neuro-metabolic/vascular coupling)^52,53^. Our data suggest that the metabolic compartmentalization necessary for neuron-astrocyte cooperation^52,53^ may be, at least partially, present early along differentiation starting from stem/progenitor cells.

Although considerable storage of Ace and Lac is classically linked to metabolic pathways inside astrocytes^52,53^, our data suggest that they are also fundamental for both NSC and OEC maintenance, probably in relation to their key role in preserving SC state and homeostasis^14^.

The shuttle of branched amino acids/ketoacids between astrocytes and neurons appears fundamental for replenishing brain Glu reserves^55^ in conjunction with Ace astroglial trafficking^56^. As detected by both ^13^C-NMR^41^ and *in vivo* ^1^H-[^13^C] magnetic resonance spectroscopy (MRS)^57^, the cell specific Ace uptake activates a glial metabolic pathway ending with the production of glutamine which can be transported to glutamatergic and GABAergic neurons for replenishing the neurotransmitter Glu (Glu-glutamine cycle) after synaptic transmission^58,59^. Glutamate is the major excitatory neurotransmitter of the brain which connects carbohydrate and amino acid metabolism via the tricarboxylic acid (TCA) cycle^52,53^ and it may act as a regulator of adult neurogenesis/differentiation^60,61^. Interestingly, we retrieved that Glu content is significantly increased in AD NSCs probably in relation to the crosstalk between neurogenesis and neurotransmitter signaling^62,63^ required for their “on demand” activation. In the adult niche NSCs are quiescent, but require rapid proliferation followed by differentiation to guarantee tissue homeostasis and renewal, especially in the presence of brain injury/damage^24^. Therefore, it is not surprising that AD NSCs possess peculiar hallmarks different from both OECs and their embryonic/postnatal counterparts, such as the highest Glu content. Moreover, we retrieved relevant and comparable levels of Glu and Suc in OECs and in the younger NSC types (E12, P1) whereas AD NSCs contained the highest (statistically significant) metabolite amounts. Concordantly, the key role of Suc and other molecules related to Krebs cycle (such as Glu) on stemness has been demonstrated in the physiological proliferation of AD NSCs^64^ coupled to their decrease in a human immortalized striatal neural SC line along differentiation^65^.

The retrieved comparable intracellular Lac content between AD and OEC samples is supported by literature data on the limited *in vivo* assignation of a specific glycolytic flux to any brain cell types by NMR spectroscopy^59^. Our data suggest that relevant Lac content is equally fundamental for the preservation of both NSCs and glial committed OECs, probably in relation to its involvement in antioxidant defense and SC homeostasis. Concordantly, metabolism of pluripotent and multipotent stem cells depends on glycolysis with hyperlactate production^45^. As matter of fact, all NSCs showed lower, but comparable content for Lac, indicative of a highly glycolytic metabolism, as already demonstrated in human NSCs^66^, whereas a decrease in Lac content has been already linked to the process of NSC differentiation^45^. Concordantly, astrocytes store higher Lac amounts as energy supply to the high energy demanding neurons of surrounding areas via a specialized shuttle transport^67^, as our OECs statistically did in comparison to E12/P1 NSCs.

Alanine is a highly gluconeogenic amino acid and was retrieved among more representative metabolites only in P1 OECs encompassing almost 40% of their main analyzed metabolites along with some aromatic amino acids, which specifically characterize OECs. Alanine appears involved in a variety of brain biological pathways including glycolysis, fatty acid and amino acid metabolism, citrate and urea cycle as well as Tau and hypotaurine metabolism^41,68^. This latter metabolic pathway is linked to the astrocyte specific antioxidant and neuroprotective capability to contrast endogenous ROS^52,53^. Moreover, similarly to astrocytes, the metabolic content in OECs suggests a possible active role of these cells also in neuromodulation as well as neurotransmitter synthesis and function. Altogether our data indicated that OECs and astrocytes appeared to share several metabolic hallmarks and may be equally able to support neuronal activity, but in different localization of the nervous system^27,38^. Concordantly to our observations, a developmental differences in Glu metabolism and neurotransmitter synthesis between postnatal and adult brain has been reported^69^.

Interestingly our analysis on brain SC/progenitor cells also enlightened an overlapping metabolic pathway redundancy able to guarantee a fine tuning of proliferation combined to the highest antioxidant defense.

Contrary to the literature, we did not observed NMR-detectable mobile lipids^17^ nor apoptotic peaks^70^, but these discrepancies may be related to the procedures for sample preparation. Additionally, NSCs are characterized by very low intensity or absent lipid signals that are instead more frequently observed in cancer SCs^43^.

Altogether, the retrieved data suggest that several aspects of metabolism may distinguish NSCs from their differentiated counterparts (OECs). All the metabolites contained in OECs are functionalized to their supportive role to the surrounding neurons whereas the NSC differential activities, at least between fetal/postnatal and adult life, are mirrored by their metabolomes. Apparently, OECs and NSCs present a univocal characteristic metabolic signature, able to determinate their fate and properties, as already demonstrated for adult NSCs^71^. This tight regulation appears preserved even when cells are induced to proliferate and maintained under artificial culture conditions. Our findings also suggest that multivariate analyses of NMR spectra may be exploited as an effective tool for the selective (SC) identification in addition to the standard immuno-biochemical investigations.

Mapping the physiological or pathological neurochemical profile of SCs by NMR may represent an invaluable tool readily transferable from research to therapeutic clinical interventions. Interestingly, as recently highlighted by Gebregiworgis and Powers^18^, in a decade of failures of proteomics^72^, NMR metabolomics might turn out to also become an efficient, alternative tool in the search of human (disease) specific biomarkers.

Overall our data provide a detailed description of the metabolic fingerprinting of cultured brain cell subtypes which deserves to be further validated and deepened. The observed metabolome appears to be unaffected by extensive cell culturing and correlates to the specific tissue derivation, (stem) characteristics and properties. Our study also shows that NMR analysis may efficiently characterize AD NSCs, otherwise indistinguishable from embryonic/postnatal NSCs. Here we also provide a detailed metabolic characterization of cultured OECs which, at the best of our knowledge, has never been previously described. Therefore, the unambiguous clustering of NMR data either by quantitative or multivariate analysis could define the physiologic homeostasis and properties of the SC pool in different environments or describe their alterations in pathological conditions (such as ageing and neurodegeneration). Importantly, such strategy requires cooperation and integration among several methodologies and fields of expertise in order to provide a better description of biological features translatable into novel methodological or therapeutic approaches.

## Materials and Methods

### Availability of materials and data

All data generated or analyzed during this study are included in this published article (and into the related Supplementary Information files).

### Animals

Experiments were performed on CD1 mice obtained by Charles River Laboratories Italy). Animals were kept in a controlled environment (23 ± 1 °C, 50 ± 5% humidity) with a 12 h light/dark cycle with food and water available ad libitum. Experiments were performed in compliance with the Italian law and in accordance with the European Community Council Directive (86/609/EEC). All the experimental procedures were carried out and authorized in accordance with Italian Law 26/2014. The research protocol was approved by the ethics committee of IRCCS Istituto Auxologico Italiano. Animals were kept at the dedicated Animal Facility afferent to our Institute wherein we performed animal sacrifices at the specified time points. All possible efforts were made to reduce both the number of animals as well as their suffering.

### NSC derivation from SVZ

Cortexes of E12.5 embryos (8–10 littermates for each experiment) and SVZs from postnatal P1 (8–10 littermates for each experiment) as well as adult (AD) (1 animal for each experiment) were dissociated to obtain single NSCs, as schematized in Supplementary Fig. S1. They could be indefinitely grown in suspension as neurospheres in proliferative medium (DMEM/Ham’s F12 medium, Invitrogen-Thermo Fisher Scientific, Waltham, MA, USA) supplemented with B27 (Invitrogen-Thermo Fisher Scientific), 5 mg/ml heparin (Sigma-Aldrich, St. Louis, MO, USA), plus mitogens: 20 ng/ml basic Fibroblast Growth Factor and 50 ng/ml Epidermal Growth Factor (both from Peprotech, Rocky Hill, NJ, USA)^73^. All cultures were analyzed in triplicate and 5 different cultures for each SC type were performed.

### Primary OB-derived OEC cultures

Primary OEC cultures were obtained from the olfactory bulbs of postnatal mice (P1, 8–10 littermates for each experiment), which were dissociated, as previously described^74^. OECs were cultured in DMEM (high glucose) (Invitrogen-Thermo Fisher Scientific) +10% Fetal Bovine Serum (FBS, Sigma-Aldrich), 2 mM L-glutamine, penicillin (50 U/ml) and streptomycin (50 µg/ml), all from Invitrogen-Thermo Fisher Scientific. All cultures were analyzed in triplicate and 3 different cultures for each SC type were performed.

Stemness potential and differentiative capability of our cells were previously tested according to published literature^24,26,73^.

### Preparation of SC lysates for NMR analyses

The same amount of cells was analyzed (3 × 10^6^ cells/SC type). Adherent OECs (3 × 10^6^ cells/SC type) were then harvested by trypsin digestion (as previously detailed^74^) while free-floating NSCs were collected by simple centrifugation, 200 relative centrifugal force (rcf), and rinsed with 5 ml of Phosphate-Buffered Saline buffer (PBS, Sigma-Aldrich). Upon centrifugation at 4,000 rcf for 1 min, cell pellets were kept on ice for 5 min before being resuspended in 1 ml of ice-cold 50% acetonitrile (VWR, Radnor, PA, USA) in PBS. Cell suspensions were kept on ice for 10 min before centrifugation at 16,000 rcf for 10 min at 4 °C. The aqueous acetonitrile extract method was chosen in accord with previous literature^28^. A total number of 51 samples (12 samples for each NSC type and 15 samples for OECs) were analyzed.

### NMR Spectroscopy

#### General

The aqueous acetonitrile extract solutions were dried down by a suitable concentrator (Savant SpeedVac®Concentrator, Thermo Fisher Scientific) and the resulting white residue was dissolved in 60 µl of PBS-Deuterium Oxide (PBS-D_2_O, as detailed below) stock solution buffered at pH = 7.0. The solution is further diluted in 540 µl of D_2_O containing trimethylsilyl-3-propionic acid (TSP, Sigma Aldrich) 1 mM as internal standard and then transferred in a 5 mm NMR tube. The PBS-D_2_O stock solution was obtained by dissolving a PBS tablet (Sigma-Aldrich) in D_2_O (Sigma-Aldrich) at a concentrations of 9.8 mg/ml.

All the NMR spectra were recorded on a Bruker Avance 500 spectrometer, Bremen Germany, operating at 500.13 MHz proton frequency and equipped with a QNP four-nuclei switchable probe. Standard ^1^H-NMR spectra acquisition were performed using residual water presaturation pulse sequence, spectral window SW = 5400 Hz, relaxation delay D1 = 4 s, 32 K points for acquisition, 512 scans. The two-dimensional TOtal Correlation SpectroscopY experiments (2D-TOCSY) were acquired with mixing time of 200 ms, SW = 5400 Hz, D1 = 4 s, 2k data points in F2 dimension and 512 FIDs in the F1 dimension. For all the experiments, the temperature was kept constant at 305 K.

All the one-dimensional NMR spectra were phase-corrected manually, while the baseline correction was performed automatically with a fifth order polynomial fitting routine. TSP was used as internal reference standard (0 ppm) for spectra calibration. The peak area was determined manually for each resolved peak representative of a single metabolite and then normalized respect to the TSP signal area. The final metabolite concentration is expressed as mM and reported in Fig. 2 for each cell line.

The assignment of the metabolite signals for each cell line was supported by the use of two-dimensional correlation NMR methods. In particular, 2D total correlation spectroscopy (TOCSY) experiments were extensively used.

### Multivariate analyses: PCA and PLS-DA

The first step in order to carry out PCA and PLS-DA is the so called “spectral binning”. After the NMR data processing described in the NMR section, spectral binning was performed on the 1D spectra by reducing the spectrum data points (32 k points) into a smaller number of segments (bins) of a defined width (0.04 ppm). The spectral area within each bin is then integrated to yield a vector that contains intensity based descriptors of the original spectrum. Binning was performed using MNova 6.0 software package (Mestrelab Research, Santiago de Compostela – Spain).

The data were then imported into Metaboanalyst 3.0, an online tool freely available at www.metaboanalyst.ca ^75^. PCA and PLS-DA were performed on a wide spectral region, 0.5–9.5 ppm (the region from 4.5 to 5 ppm was blocked out due to water suppression artifact). Normalization for all of the spectral binning data is performed on the total area of the NMR spectrum. This is achieved by calculating the sum of all the variables within a spectrum and by normalizing each spectrum on such value. In this way, every single variable is converted to a fraction of the total spectral area or intensity. Pareto scaling is then applied. Accordingly, the variable mean was subtracted from each variable (column of the data) and then each variable was divided through the square root of its standard deviation. The raw data were then exported into Origin 9.0 in order to create three-dimensional graphs.

PLS-DA is a supervised mathematical method able to enhance the cluster separation already obtained with the PCA method. For PLS-DA, the best linear combination of the original PCs are used as new variables. The results are shown in Fig. 4. Cross-validation was achieved by the leave-one-out validation protocol followed by the permutation test (n = 100).

### Quantification of extracellular Lac levels

Analysis was conducted by a kit (Glycolysis Cell Based Assay Kit Cayman Chemical Company, Ann Arbor, MI, USA) for the quantification of extracellular L-Lac levels (the end product of glycolysis) which are proportionally correlated with intra-cellular glycolytic activity. Briefly, cells deriving from 2 different cultures for each NSC type (15.000 cells/well) were plated in duplicate in 96 wells and cultured for 60 hours. Afterwards, standards and samples (10 µl of cell conditioned media as well as an equal amount of cell-free media, NSC and OEC medium) were processed and analyzed following the manufacturer’s instructions. L-Lac levels were calculated subtracting the absorbance levels of the corresponding cell-free media and corrected for the total cell number in each well.

### Determination of Glc content and uptake from media

Quantitative analysis of Glucose (Glc) content in media was performed on 0.5 ml of NSC/OEC medium. Cells (25.000 cells/ml) were grown for the indicated times (5 or 7 Days In Vitro, DIV) and thereafter their media were collected and analyzed by NMR. Concentrations of Glc were calculated as difference between their value in naïve medium and the retrieved amount after cell growth. Each sample was prepared in triplicate and all data were normalized for the total cell number.

### Statistics

Each experiment was run in triplicate and representative values were expressed as mean ± SD. Data were analyzed using One way ANOVA analysis followed by uncorrected Fisher’s LSD (the corresponding p is specified in the respective figure or table), using a dedicated statistical software (GraphPad Prism, Inc., La Jolla, CA, USA). Metabolic pathways were investigated by the KEGG PATHWAY Database – GenomeNet, free available online at: www.genome.jp/kegg/pathway.html.

## Electronic supplementary material

Supplementary Information
