# Supplementary material for: NMR Metabolomics for Stem Cell type discrimination
Source: Sci Rep. 2017 Nov 17;7:15808. doi: 10.1038/s41598-017-16043-8 (PMC5693937; doi:10.1038/s41598-017-16043-8)

Supplementary information to manuscript "NMR Metabolomics for Stem Cell type discrimination"

Franca Castiglione [a¥]; Monica Ferro [a¥]; Evangelos Mavroudakis [a,b¥]; Rosalia Pellitteri [c]; Patrizia Bossolasco [d]; Damiano Zaccheo [e]; Massimo Morbidelli [f]; Vincenzo Silani [d,g]; Andrea Mele [a,h]; Davide Moscatelli [a§] and Lidia Cova [c§*]

a. Dept. of Chemistry, Materials and Chemical Engineering G. Natta, Politecnico di Milano, Milan, Italy.

b. Current address: Environmental Research Laboratory, National Center for Scientific Research "Demokritos", Agia Paraskevi Attikis 15310, Greece

c. Inst. of Neurological Sciences, CNR, Section of Catania, Catania, Italy.

d. Dept. of Neurology and Lab. Neuroscience, IRCCS Istituto Auxologico Italiano, Milan, Italy.

e. Dept. of Experimental Medicine, section of Human Anatomy, University of Genoa, Genoa, Italy.

f. Inst. for Chemical and Bioengineering, ETH Zurich, Zurich, Switzerland.

g. Dept. Pathophysiology and Transplantation -"Dino Ferrari" Center, Università degli Studi di Milano, Milan, Italy.

h. ICRM Istituto di Chimica del Riconoscimento Molecolare, CNR, Milan, Italy.

§ co-senior-author

*Corresponding author

Supplementary Results, Legends and Bibliography

The metabolic content of *in vitro* highly proliferating SC types correlates to their physiological function and identity

As clearly depicted in Supplementary Fig. SI1A, our four cell subtypes derive from two correlated regions of the mouse brain (respectively the Olfactory Bulb, OB, and the Subventricular Zone, SVZ). The cartoon illustrated also the specific isolation and maintenance of the cell subtypes *in vitro* (Supplementary Fig. SI1B) for several passages, as well as a representative figure of the cultured cell sharing the neural stem/progenitor cell marker Nestin (Supplementary Fig. S1C). Supplementary Fig. S2 exemplified a contour plot of ^{1}H TOCSY for the identification of protons on sugar rings or amino acids. Supplementary Figs. SI3-5 clearly illustrated the qualitative/quantitative differences in metabolic content and the related metabolic pathways among the four cell subtypes in culture which can be related to their physiological functions, as detailed in Discussion. The ^{1}H-NMR spectra resulting from intracellular analysis allowed to distinguish the peaks of metabolites characteristic of highly proliferating cell populations, such as Cho in conjunction with different amino acids (Suc, Glu, Ala) and Lac as the end product of anaerobic glycolysis. As a matter of fact, extracellular Lac, a product from glucose metabolism, derives from pyruvate conversion and it is indirectly indicative of the overall glycolysis rate. Firstly, we compared this metabolite value in NSC conditioned media by a specific Assay Kit seeking possible metabolic alterations due to development and/or ageing, but no significant differences among samples were retrieved (Supplementary Fig. SI7A). Therefore we decided to study the cell consumption of Glc from media (see Supplementary Fig. SI7B) in a time-course (5 and 7 days *in vitro*, DIV). As expected, we detected different Glc levels in the control media (i.e. in the absence of cells) among OEC and NSC specific media (ratio Glc content 1:3) due to their different composition (see Supplementary Fig. SI7C). Interestingly, a statistical difference among P1/AD NSCs and OECs was also retrieved for Glc consuption in media at 7 DIV, thus supporting different metabolic requests among cell subtypes. As reported in literature[1], highly proliferative cells, such as tumors and pluripotent SCs, are characterized by an anaerobic glycolytic phenotype which is also essential to maintain stemness and contrast senescence. In this complex scenario we observed a higher overall extracellular Lac concentration among NSCs irrespectively of developmental stage or ageing, supporting the

hypothesis of a conserved energy balance among embryonic, postnatal and adult NSCs, at least in culture. Finally, it is worth of mention that, according to Suppl Figs SI3,4 the Glc content in OECs was negligible in comparison to NSCs. The lowest Glc levels retrieved in OECs may derive from its immediate transformation in Lac eventually available for oxidation in mitochondria and/or release in the surroundings[2], since these cells act as physiological blood glucose sensor cells to inter-connect peripheral metabolism and brain homeostasis[3].

Legends of Supplementary Figures

Supplementary Fig. 1. Origin and function of OECs and NSCs. (A) Physiologically OECs are glial cells located in the Olfactory Bulb (OB) and they ensheath the axons of the olfactory receptor neurons. NSCs reside in the ependymal layer or Subventricular Zone (SVZ) of the lateral ventricle (LV) and have the capacity to self-renew and produce glial and neuronal differentiated cells. In the adult OB, neurons (N) and glia (astrocytes (A), as well as oligodendrocytes (O)) are replaced continuously by the NSCs, which migrate towards OB along the rostral migratory stream (RMS), therefore these cells share a common origin. Str= striatum; EPC=Ependymal cells. (B) Our samples derived from different developmental times (for NSCs: embryonic day 14 (E12), Postnatal day 1 (P1) and adult (AD); for OECs: Postnatal day 1 (P1)) and correlated brain regions (SVZ for NSCs and OB for OECs). All samples were then analyzed by NMR. (C) Representative confocal images of OECs and NSCs (scale bar 30 μm).

Supplementary Fig. 2. Stacked plot of ^{1}H NMR spectra of cell lysates. Peak assignment is referred to those specific signals of each metabolite suitable for integration (see text for abbreviations). The cells sub-types are in the following order: E12 NSCs from SVZ at embryonic day 12; P1 NSCs from SVZ at postnatal day 1; OEC Olfactory Ensheating Cells from olfactory Bulb (OB) of postnatal day 1 mice; AD NSCs from adult mouse SVZ.

Supplementary Fig. 3 Aliphatic region of ^{1}H TOCSY experiment performed on the E12 NSCs. The assignment of the most significant metabolites is also shown. In a typical TOCSY

contour plot, the spectral peaks can be grouped in diagonal and off-diagonal peak. The latter are commonly referred to as cross-peaks. Each cross-peak connects two spectral signals generated by H nuclei with spin-spin coupling. Thus this experiment allows to reconstruct the spin systems associated to a given molecular fragment, and supports the spectral assignment.

Supplementary Fig. 4. Metabolites concentration (expressed in mM) for all SC types. The x-axis reports the type of metabolite observed and quantified by ^{1}H-NMR spectroscopy. The metabolites level (mM concentration) detected is reported on the y-axis. Different color bars (see legend) are used for each SC type. Error bars are referred to the standard error.

Supplementary Fig. 5. Kegg pathway analysis for the retrieved metabolites. Correlations among more representative metabolites (74% of the nine examined metabolites/each cell type) and the main involved biological cascades retrieved in the Kegg database. Noticeably, several pathways are shared/common (highlighted with the same colors), but some are specifically related to the biological functions exerted by cell populations, as detailed in the main text.

Supplementary Fig. 6. Analysis of metabolite content in brain SCs. No statistically significant differences among metabolite concentrations were retrieved among the cell content of Choline (Cho), Alanine (Ala), Acetate (Ace), Creatine (Cr) and Glucose (Glc) in different SC types. Cell lysates were obtained from 25.000 cells/ml grown in the appropriate medium and resuspended in PBS-D_2O stock solution. Data are expressed as metabolite concentrations calculated on the intensities of the integral of the peaks retrieved by ^{1}H-NMR spectroscopy. One way ANOVA followed by uncorrected Fisher's LSD was performed, as specified in the statistical section.

Supplementary Fig. 7. Overall glycolysis rate is conserved among NSCs, but Glc content and consumption is different among SC types, as demonstrated by quantification of extracellular Lac levels. (A) A specific assay (a colorimetric method for detecting extracellular L-lactate, the end product of glycolysis, in cultured cells) was performed to study eventual

metabolic differences among NSCs after 60 hours in culture. Data are reported as extra-cellular Lactic Acid level (mM) normalized on cell number (mM/cell) and comparable glycolytic rate were observed. (B) and (C) Cells were grown for the indicated times (5 or 7 Days in vitro, DIV) and thereafter their media were collected and analyzed by NMR. Concentration of Glc (b) were calculated as difference among their value in naïve medium and the retrieved amount after cell growth. Therefore, higher values depended on massive Glc consumption. Data are expressed as mean of metabolite concentrations ± SEM, calculated on the intensities of the integral of the peaks retrieved by ^{1}H-NMR spectroscopy on cell media and normalized for cell number. Statistical significance is indicated in each graph, if retrieved, as $*p \leq 0.05$ or $**pp \leq 0.01$ *vs* the corresponding sample(s). (c) Quantitative analysis of Glc content in basal NSC and OEC media. Higher Glc content was retrieved in NSC *vs* OEC medium in a ratio around 1:3 since NSCs require higher Glc levels in medium than OEC cultures. One way ANOVA followed by uncorrected Fisher's LSD was performed, as specified in the statistical section.

Supplementary Fig. 8. 3D Score plot of PCA analysis. The 3D score plot enlightened the similarities and differences among the metabolic fingerprints of each SC type –PCA score plot of PC1 vs PC2 vs PC3 for: E12, P1, AD, and OEC SCs. The data points correspond to the examined samples. The proximity of two points reflects the metabolic similarity among the corresponding samples. The grouping of each cell type is also outlined by the color code of the points confined in the ellipsoids. Clustering and separation of the points related to OEC is clearly indicated by the blue ellipsoid. For the presented analysis, the entire ^{1}H NMR spectrum was divided in 0.04 ppm regions (bins) for each sample.

Supplementary Fig. 9. 2D Projection in the PC1-PC2 plane of the score plot of PCA and PLS-DA analyses. (A) The plot is the projection in the PC1-PC2 plane of the data of Supplementary Figure SI8. (B) 2D Projection in the PC1-PC2 plane of the score plot of PLS-DA analysis. The plot is the projection in the PC1-PC2 plane of the data of Fig. 4 of the manuscript. The tags indicate the type of SCs associated to the datasets. Symbol legend: X=E12, +=P1, Δ=AD, ◊= OEC.

Supplementary Fig. 10. 2D Score plots of PCA (A) and PLS-DA (B) analysis carried out on NSC subset. The 2D score plots do not show appreciable cluster separation within NSC types. The tags indicate the type of NSCs associated to the datasets. Symbol legend: x=E12, +=P1, Δ=AD.

Supplementary Fig. 11. Permutation test of PLSDA analysis: the iteration number is 100. (A): Permutation test on data of Fig. SI8. (B): Permutation test on data of Fig. SI11.

Bibliography

1. Ito, K. & Suda, T. Metabolic requirements for the maintenance of self-renewing stem cells. *Nat. Rev. Mol. Cell Biol.* **15,** 243–56 (2014).

2. Passarella, S. *et al.* Mitochondria and l-lactate metabolism. *FEBS Letters* **582,** 3569–3576 (2008).

3. Al Koborssy, Al Koborssy, D. *et al.* Cellular and molecular cues of glucose sensing in the rat olfactory bulb. *Front. Neurosci.* **8,** (2014).

Supplementary Figure SI1

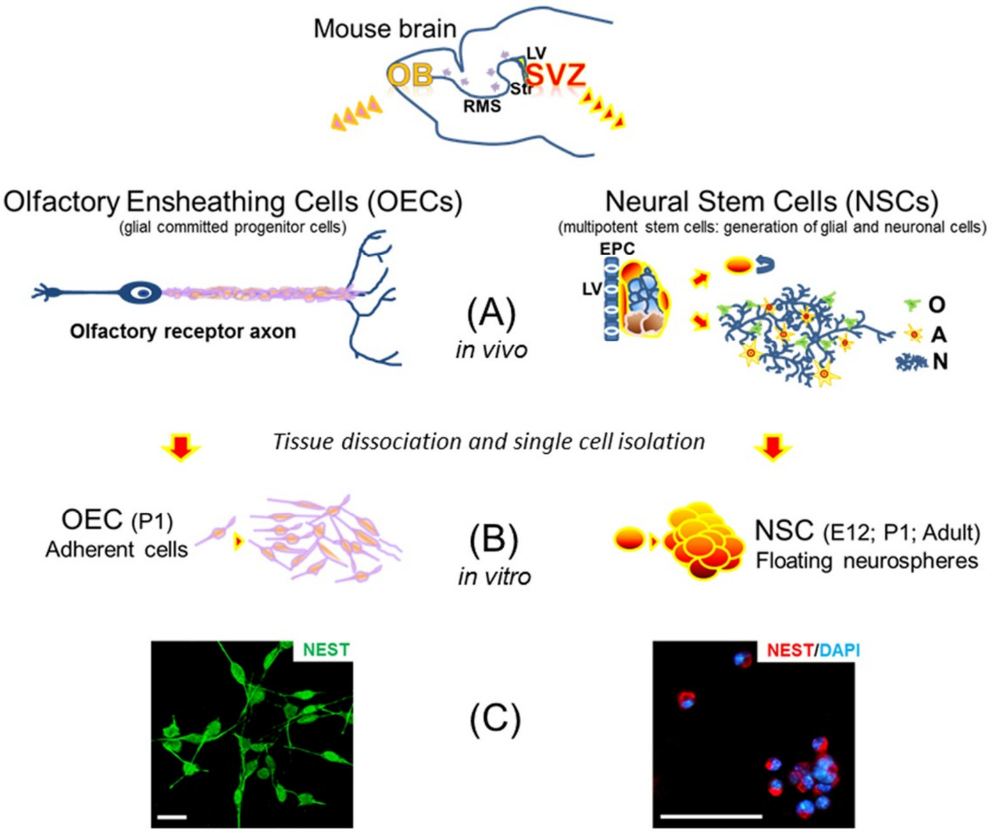

Supplementary Figure SI2

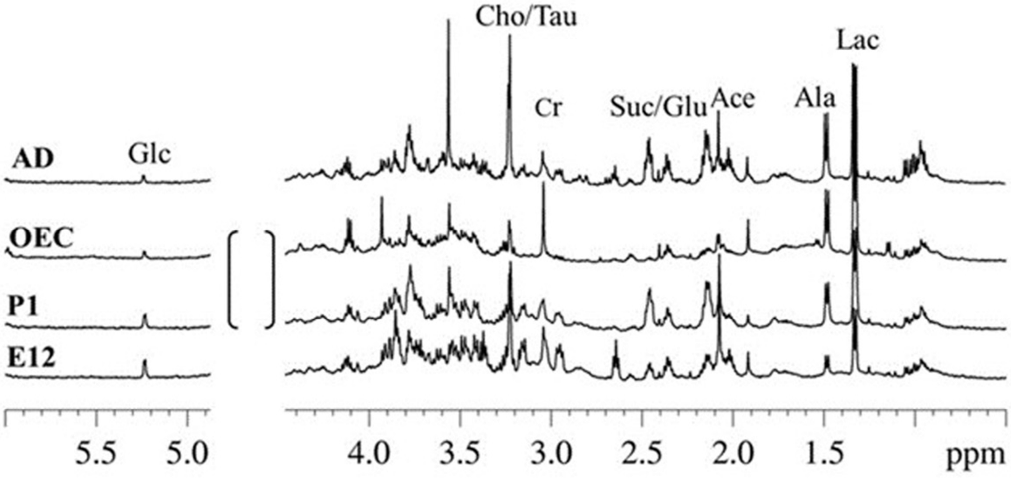

Supplementary Figure SI3

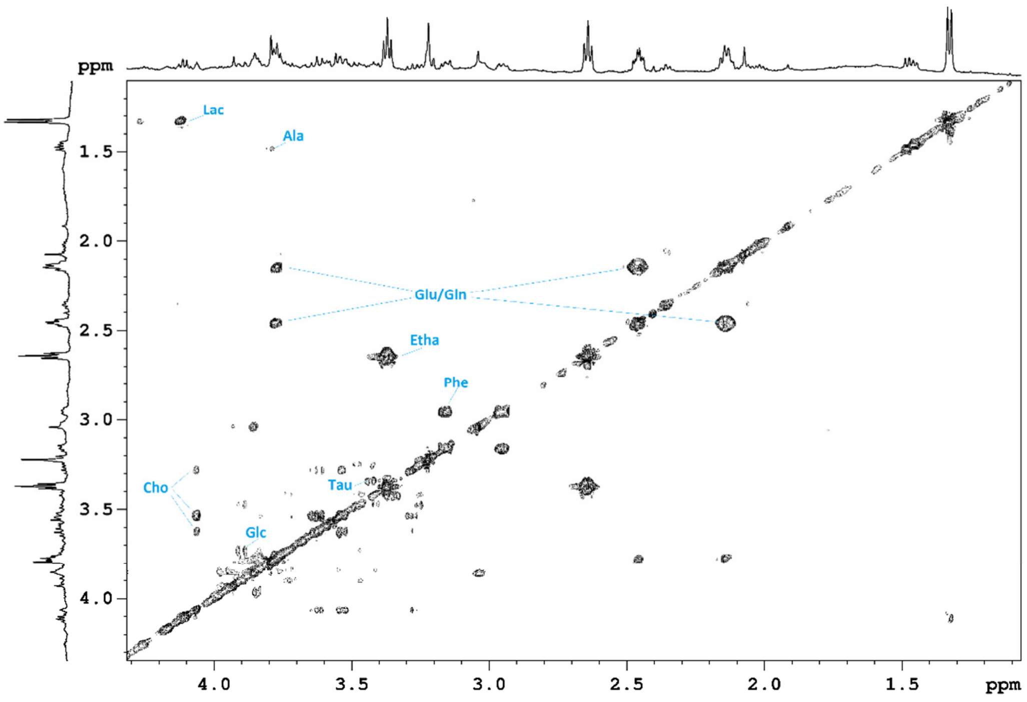

Supplementary Figure SI4

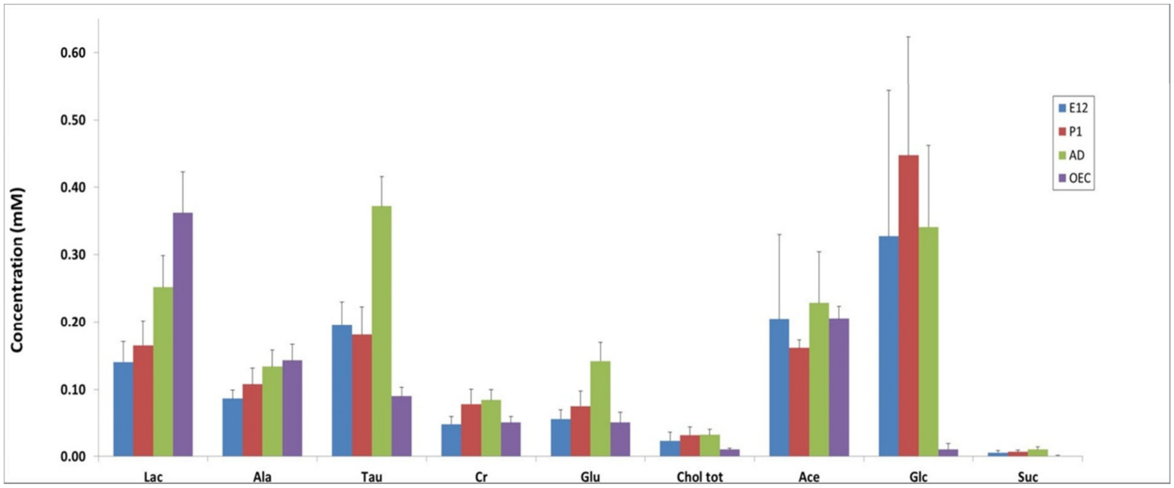

Supplementary Figure SI5

GLC (only NSCs):

2-Oxocarboxylic acid metabolism
ABC transporters
Adipocytokine signaling pathway
AGE-RAGE signaling in diabetic complications
AMPK signaling pathway
Ascorbate and aldarate metabolism
Autophagy
Central carbon metabolism in cancer
FoxO signaling pathway
Fructose and mannose metabolism
Galactose metabolism
Glycolisis/Gluconeogenesis
Glycerolipid metabolism
Glucagon signaling pathway
HIF-1 signaling pathway
Insulin secretion and metabolism+resistance
Longevity regulating pathway
Mucin type O-glycan+other types of O-glycan biosynthesis
Neuroactive ligand-receptor interaction
Oxytocin signaling pathway
Pentose phosphate pathway
PI3K-Akt signaling pathway
Pyrimidine metabolism
PPAR signaling pathway
Prolactin signaling pathway
Thyroid hormone synthesis+signaling pathways

LAC (both NSCs and OECs):

2-Oxocarboxylic acid metabolism
Butanoate metabolism
Central carbon metabolism in cancer
Cysteine and methionine metabolism§
Fructose and mannose metabolism
Glucagon signaling pathway
Glycolisis/Gluconeogenesis
Histidine metabolism*
HIF-1 signaling pathway
Pantothenate and CoA biosynthesis
Phenylalanine metabolism*
Pyruvate metabolism
Propanoate metabolism
Tyrosine metabolism*
Tryptophan metabolism*
Ubiquinone and other terpenoid–quinone biosynthesis
Valine, leucine and isoleucine biosynthesis

TAU (only NSCs)§:

ABC transporters
Cysteine and methionine metabolism§
Glutathione metabolism
Neuroactive ligand-receptor interaction
Sulfur metabolism

ACE (both NSCs and OECs):

2-Oxocarboxylic acid metabolism
Alanine, aspartate and glutamate metabolism
Alpha linoleic acid metabolism
Arginine and proline metabolism
Butanoate metabolism
Cholinergic synapse
Central carbon metabolism in cancer
Citrate cycle (TCA cycle)
Dopaminergic synapse
Drug metabolism cytochrome p450
Glycolisis/Gluconeogenesis
Glycine, serine and thereonine metabolism
Glycosaminoglycan biosynthesis/heparan sulphate/heparin
Glyoxylate and dicarboxylate metabolism
Glucagon signaling pathway
Lysine degradation
Histidine metabolism*
Metabolism of xenobiotics by cytochrome P450
Phenylalanine metabolism*
Pyruvate metabolism
Propanoate metabolism
Serotoninergic synapse
Synthesis and degradation of ketone bodies
Sulfur metabolism
Taurine and hypotaurine metabolism
Terpenoid backbone biosynthesis
Tyrosine metabolism*
Tryptophan metabolism
Valine, leucine and isoleucine degradation

ALA (only OEC):

2-Oxocarboxylic acid metabolism
ABC transporters
Alanine, aspartate and glutamate metabolism
Arginine biosynthesis
Arginine and proline metabolism
Beta-alanine metabolism
Butanoate metabolism
Central carbon metabolism in cancer
Citrate cycle (TCA cycle)
Cysteine and methionine metabolism§
D-glutamine and D-glutamate metabolism
Fatty acid biosynthesis and degradation
Dopaminergic synapse
Folate biosynthesis
GABAergic synapse
Glyoxylate and dicarboxylate metabolism
Glycine, serine and thereonine metabolism
Glutathione metabolism
Histidine metabolism*
Lysine biosynthesis
Neuroactive ligand-receptor interaction
Nitrogen metabolism
Pantothenate and CoA biosynthesis
Phenylalanine metabolism*
Phenylalanine, tyrosine and tryptophan biosynthesis*
Pyrimidine metabolism
Prolactin signaling pathway
Propanoate metabolism
Purine metabolism
Selenocompound metabolism
Sulphur relay system
Taurine and hypotaurine metabolism§
Thiamine metabolism
Tyrosine metabolism*
Tryptophan metabolism*
Ubiquinone and other terpenoid–quinone biosynthesis

* Aromatic amino acids
§ Sulphur containing aminoacids

Supplementary Figure SI6

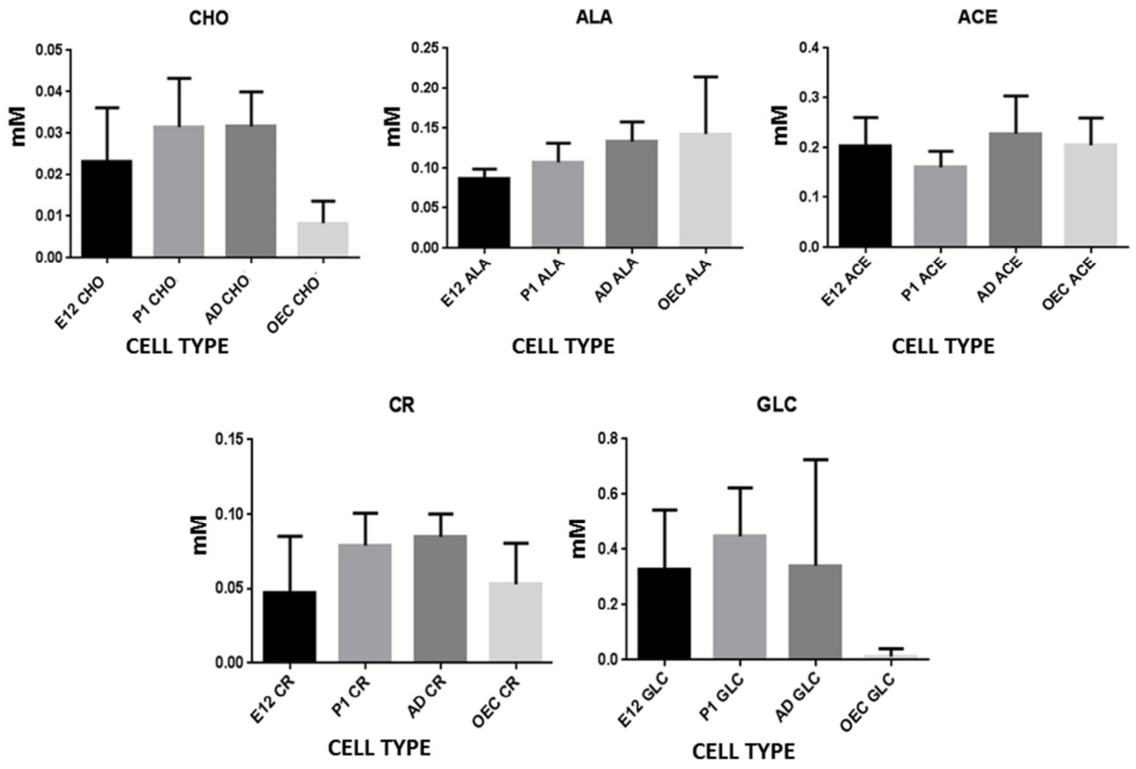

Supplementary Figure SI7

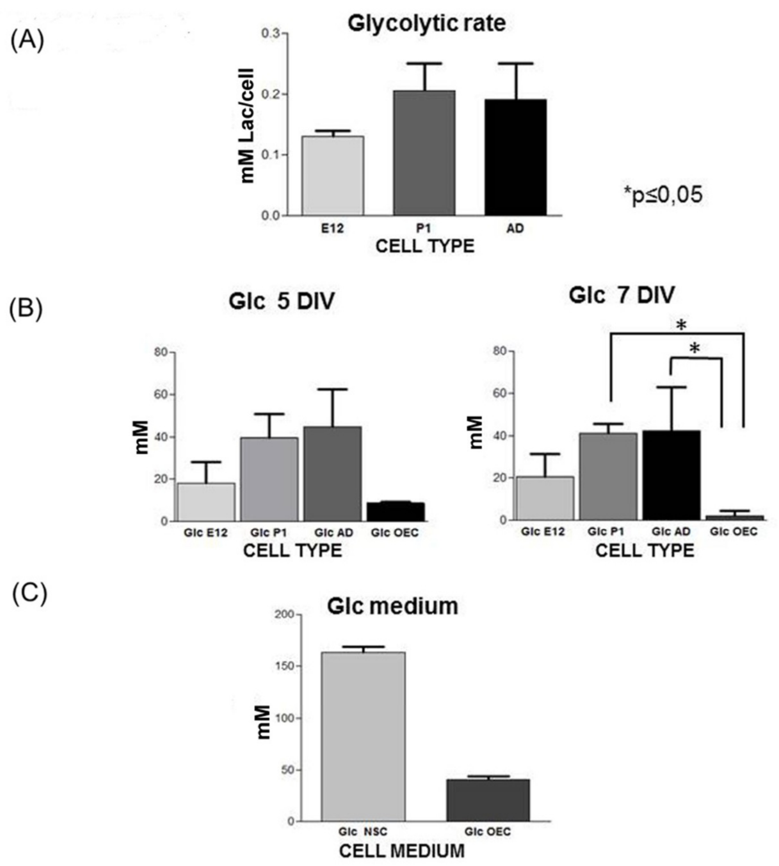

Supplementary Figure SI8

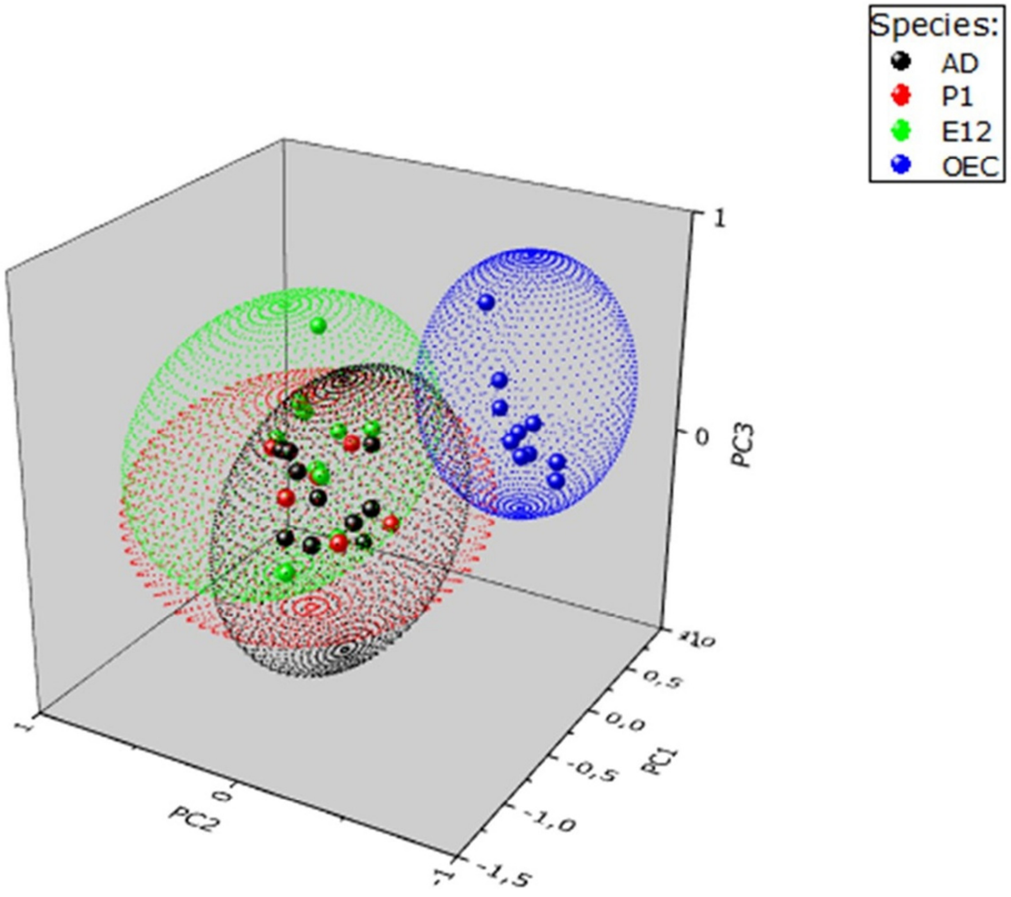

Supplementary Figure SI9

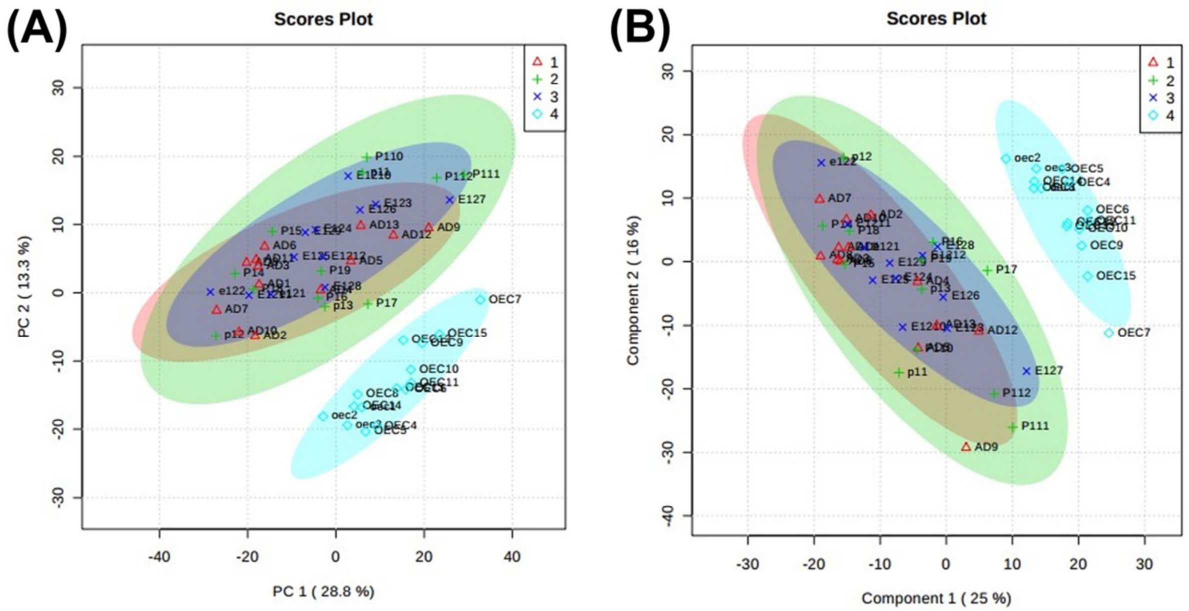

Supplementary Figure SI10

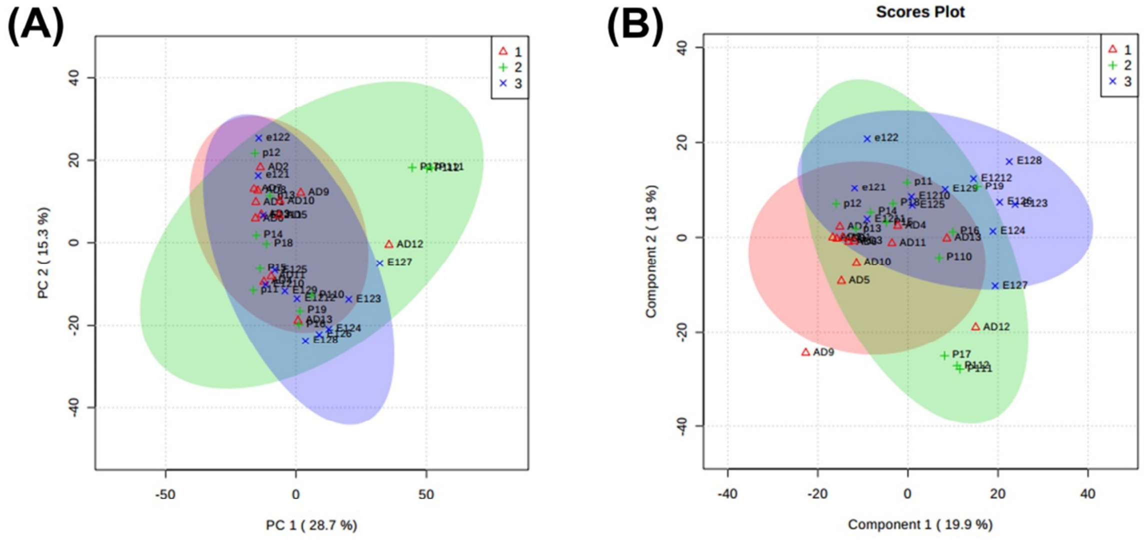

Supplementary Figure SI11

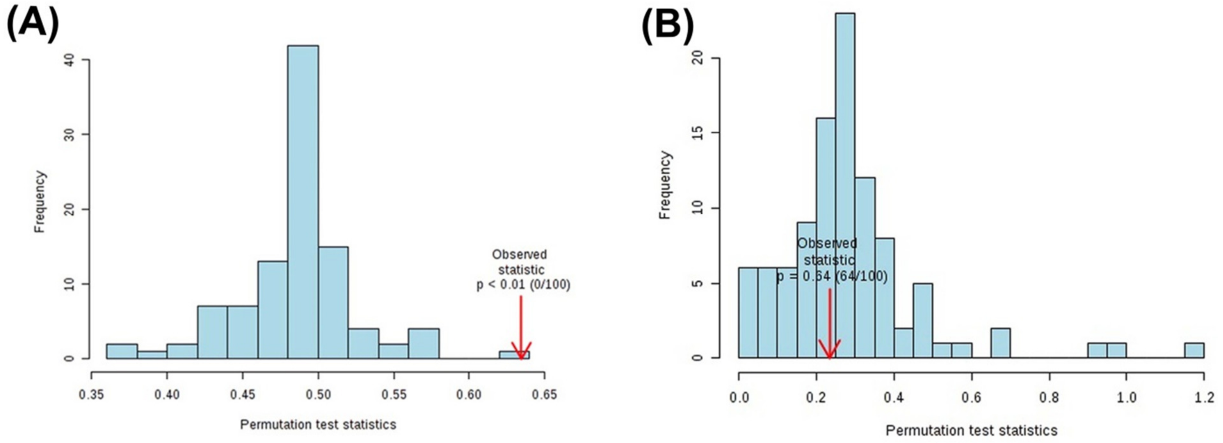

Supplement: Supplementary file 1 — Supplementary Information [file 41598_2017_16043_MOESM1_ESM.pdf]
